# Serum Cystatin C as a Biomarker in Diffuse Large B-Cell Lymphoma

**DOI:** 10.3390/scipharm85010009

**Published:** 2017-03-08

**Authors:** Nada E. Hammouda, Manal A. Salah El-Din, Mamdouh M. El-Shishtawy, Amal M. El-Gayar

**Affiliations:** 1 Department of Biochemistry, Faculty of Pharmacy, Mansoura University, Mansoura 35516, Egypt; mshisht@mans.edu.eg (M.M.E.-S.); amalgayar@mans.edu.eg (A.M.E.-G.); 2 Oncology Center, Faculty of Medicine, Mansoura University, Mansoura 35516, Egypt; manasalah@mans.edu.eg

**Keywords:** diffuse large B-cell lymphoma, cystatin C, survival, extra-nodal involvement

## Abstract

Elevated serum levels of cystatin C are found to be related to poor outcome and metastatic potential of some malignant disorders. To evaluate the clinical prominence of serum cystatin C in diffuse large B-cell lymphoma (DLBCL), blood samples were obtained from 58 patients at the time of diagnosis and paired blood samples were obtained from 22 patients at the time of remission. Also, serum cystatin C level was measured in matched healthy controls. Serum cystatin C levels were significantly more elevated in DLBCL patients than in controls (*p* < 0.0001). Furthermore, paired-sample analysis revealed that pretreatment cystatin C levels were reduced significantly in patients who achieved remission after therapy (*p* = 0.016). High serum cystatin C levels were correlated with age over 60 years (*p* = 0.049), extra-nodal involvement (*p* = 0.005) and with high serum lactate dehydrogenase (LDH) (*p* < 0.013). Elevated serum cystatin C levels were associated with extra-nodal involvement and they were significantly reduced to normal range after the remission. However, Kaplan–Meier curves revealed no survival difference in the pretreatment serum cystatin C levels. Therefore, serum cystatin C may be a novel biomarker that reflects tumor burden in DLBCL but bears no prognostic significance regarding survival.

## 1. Introduction

The furthermost prevalent subtype of non-Hodgkin lymphoma (NHL) is diffuse large B-cell lymphomas (DLBCL) found in 30%–40% of all cases of lymphoma globally [1,2]. The core tactic for treating DLBCL is multidrug combination of immunochemotherapy. Nevertheless, extended survival is reached in only half of patients, which accentuates the necessity to evolve pioneering therapeutic strategies [2,3]. Over the past few decades, investigators and medical experts have been looking to identify prognostic factors in DLBCL, so it is apparent that a new therapeutic era can begin.

Tumor biomarkers are crucial for the screening, diagnosis and follow up of cancer. Definite biomarkers for lymphoma are also relevant to the treatment plan, prognostic mapping and in studying tumorigenesis. Present-day lymphoma clinical biomarkers are non-specific and scarce. Serum lactate dehydrogenase (LDH) is an example of the most commonly used biomarker in the diagnosis of lymphoma and is correlated to prognosis [4], though it has limited specificity and clinical applications since an increased LDH serum level is also noticed in other non-neoplastic diseases, as in myocardial damage [5].

Numerous serum biomarkers for lymphomas were proposed for their clinical value such as soluble intercellular adhesion molecule-1 (s-ICAM-1/s-CD54) [6]; soluble Fas/CD95/APO-1 [7]; soluble tumor necrosis factor receptor 2 (sTNF-R2) [8]; soluble interleukin-2 receptor (sIL-2R) [9]; nm23-H1 protein [10]; and soluble CD44 [11]. Conversely, these markers are non-specific for the detection of lymphoma as they are similarly increased in other cancer types and even in non-neoplastic conditions [12,13,14]. To date, a widely adapted prognostic biomarker that is directly released from diffuse large B-tumor cells and can be easily quantified repeatedly by a simple method has not been established.

Cysteine proteinase inhibitors, cystatins, are involved in mechanisms controlling intracellular and extracellular protein degradation. Under normal physiological conditions, small amounts of catalytically active proteases, released from lysosomes or secreted from infected or dying cells, are effectively blocked by cystatins. Cystatin C, a member of the family II of cystatins, is a non-glycosylated low molecular weight basic protein, as an inhibitor of cysteine proteases, distributed pervasively in almost all extracellular fluids. [15]. A wide-ranging scale of biological significance has been advocated for cystatin C, as well as for the regulation of protein catabolism, bone resorption, inflammation, control of hormone processing, antigen presentation and T-cell dependent immune response [16,17,18].

Cystatin C has also been proposed to have a role allied to the modification of the proteolytic system in cancer. In patients with lung and colorectal cancer, elevated serum cystatin C levels are linked to meager outcome of cancer [18,19]. In patients with melanoma, increased cystatin C serum levels correlated with the stage of disease were highest for metastatic melanoma patients [20]. Thus, cystatin C level was proposed for follow up of diseases, efficacy of chemotherapy, and prediction of disease outcome. It was detected that patients with B-NHL had significantly greater cystatin C levels compared to healthy controls; the same was detected in patients with relapse of the disease when compared to patients without relapse [21].

To further our understanding of the role of cystatin C in DLBCL, we analyzed the level of cystatin C in sera of DLBCL patients and prospectively evaluated its potential influence on disease outcome.

## 2. Materials and Methods

### 2.1. Patients: Clinical Data and Treatment

We used a prospective, consecutive entry design. Between February 2014 and February 2015, 58 previously untreated patients with DLBCL, in Oncology Center, Mansoura University, Mansoura, Egypt, confirmed by biopsy, participated in this study. Patients with a creatinine level above the upper physiologic limit, patients with autoimmune diseases, asthma, and other malignant diseases have been excluded from the study. Age- and sex-matched healthy blood donors served as controls. The current study was approved by the ethical committee of the Faculty of Pharmacy, Mansoura University, Mansoura, Egypt on February 10, 2014 with code number 2016-72. All procedures implemented in this study concerning human participants were in agreement with the ethical standards of the national and institutional research committee, also in accordance with the 1964 Helsinki declaration and its late amendments.

Blood samples were collected with informed consent and institutional ethical committee approval from all patients, at the time of diagnosis, before therapy. In addition, paired serum samples were obtained at the time of disease remission in twenty-two patients just before the fifth cycle of chemotherapy. A complete response (CR) was well-defined as the disappearance of all laboratory, clinical and radiographic evidence of lymphoma. Furthermore, patients who showed response with negligible residual radiographic abnormalities were identified as partial responders (PR).

Clinical stages of the patients were categorized according to the Ann Arbor staging system [22] by means of a physical examination; biopsy; systemic computed tomography (CT) examination; bone marrow aspiration; hemogram and differential cell counts; and routine biochemical tests. Median age of the included series was 57 years (ranging from 22 to 69). Twenty-five patients (43.1%) were male. Diagnosis for all cases was according to histological benchmarks based on the classification of the Revised European-American Lymphoma/World Health Organization (REAL/WHO). The key preliminary characteristics of the patients are recorded in Table 1. Advanced Ann Arbor staging was witnessed in 50 patients (86.2%) and extra-nodal involvement in 20 patients (34.5%). Forty-eight cases had high or high to intermediate risk based upon the score of the international prognostic index (IPI) [23].

Thirty-six patients received rituximab, cyclophosphamide, Adriamycin, vincristine, and prednisolone (R-CHOP) regimen (rituximab; 375 mg/m^2^ on day 1), 19 patients received CHOP without rituximab (cyclophosphamide, 750 mg/m^2^ on day 1; Adriamycin, 50 mg/m^2^ on day 1; vincristine, 1.4 mg/m^2^ on day 1; and oral prednisolone, 100 mg daily for five days) [3], while three patients did not receive any therapy due to early death. The treatment outcome was evaluated by the revised response International Working Group criteria [24].

### 2.2. Determination of Serum Cystatin C Levels

The serum level of cystatin C in lymphoma patients was measured by specific quantitative sandwich enzyme-linked immunosorbent assay (ELISA) using Human Cystatin C Kit ELISA catalog number MBS700210 (MyBioSource, San Diego, CA, USA).

Lactate dehydrogenase was measured using ELISA kit catalog number MBS009535 (MyBioSource).

### 2.3. Statistical Analysis

Statistical analysis was performed using SPSS statistics software release 22 (SPSS Inc., Chicago, IL, USA). Quantitative data were tested for normality using the Kolmogorov–Smirnov test. Abnormally distributed data were given as median (minimum–maximum) while normally distributed data were given as mean ± standard deviation (SD). Non-parametric statistical tests of significance were applied; the Mann–Whitney U test was used to compare two independent groups and the Wilcoxon test was used to compare before/after chemotherapy. While in the case of normal distributed data, the independent Student’s *t*-test and paired match *t*-test were used instead.

Survival analysis was done using the Kaplan–Meier test. Correlations of cystatin C with clinical stages of disease, erythrocyte sedimentation rate (ESR), LDH, and IPI were defined by Spearman rank correlation analysis. All applied statistical tests of significance were two-tailed. In all tests, two-sided *p*s below 0.05 were considered significant. Receiver operating characteristic (ROC), carried out using MedCalc statistical software version 12.3 (Microsoft partner, Ostend, Belgium), was used to evaluate the diagnostic and prognostic accuracy of a test. The Youden index was used to find the cut-off point (i.e., the point that gives maximum correct classification). At this cut- off point, sensitivity and specificity were determined. Correlations were carried out using Pearson correlation (*r*) and Spearman correlation.

## 3. Results

### 3.1. Distribution of Serum Cystatin C in Patient and Control Sera

**Figure 1:**
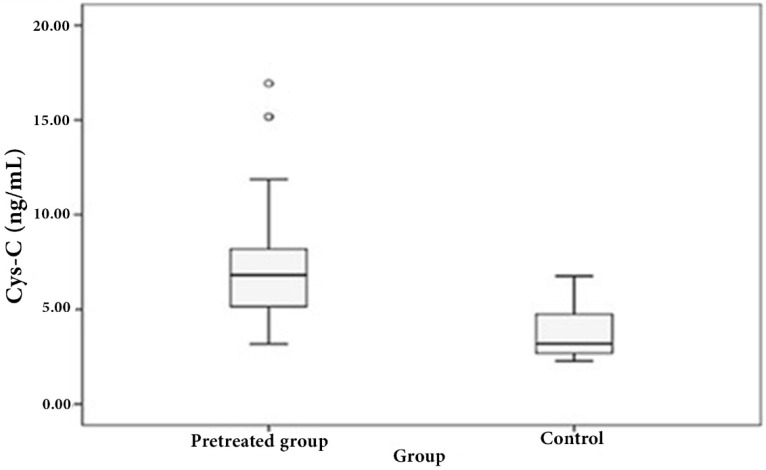
Box plot shows a comparison between the median cystatin C level (Cys-C) (ng/mL) and its range in pretreated patients versus healthy controls.

We examined the circulatory cystatin C levels of DLBCL patients. Median serum cystatin C levels of DLBCL patients at diagnosis were significantly higher than those of healthy controls (6.8091 vs. 3.1866 ng/mL, *p* < 0.0001, Figure 1).

**Figure 2:**
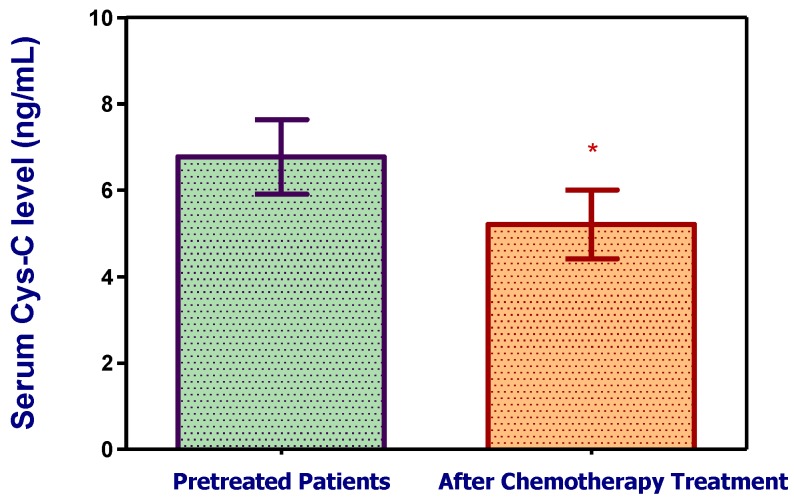
Bar chart shows the difference in the mean serum cystatin C (Cys-C) levels and 95% confidence level in the before-chemotherapy group and the after-achieving-remission group (paired group analysis of the 22 patients at the time of diagnosis and then after achieving remission). * Significant different (*p* < 0.05).

Paired-sample analysis of 22 patients revealed that cystatin C levels at diagnosis were significantly decreased at the time of remission (i.e., in the after-chemotherapy group compared to the before-chemotherapy group) (6.7728 ± 1.9435 vs 5.2098 ± 1.8025 ng/mL, *p* = 0.016; Figure 2).

In the 44 patients with DLBCL who achieved response (PR/CR) after chemotherapy treatment, initial levels of cystatin C were significantly lower (median: 5.456; range: 2.49–15.17 ng/mL) compared with the non-responders (median: 7.037; range: 3.72–10.81 ng/mL; *p* < 0.001).

### 3.2. Diagnostic Value of Cystatin C Levels in Diffuse Large B-Cell Lymphoma

**Figure 3:**
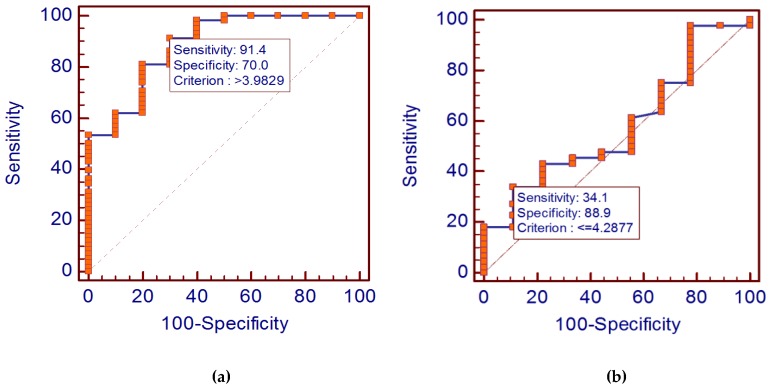
(**a**) Receiver operating characteristic curve (ROC) for serum cystatin C levels (diagnostic curve) in the fifty eight untreated group of patients; (**b**) Receiver operating characteristic curve (ROC) for serum cystatin C levels (prognostic curve comparing patients who achieve remission (responders) and patients who did not (non-responders).

To detect the diagnostic role of serum cystatin C as a biomarker in DLBCL, the ROC analysis was adopted. Analysis results suggested that: The serum cystatin C level could distinguish the DLBCL patients from the healthy individuals at an optimal cut-off point of 3.9829 ng/mL. The cystatin C diagnostic specificity and sensitivity were found to be 91.4% and 70%, respectively. Moreover, the area under curve (AUC) was 0.886 (Figure 3a).

#### 3.2.1. Relations between Serum Cystatin C Levels and Other Biological and Clinical Parameters

Levels of cystatin C in sera were correlated with various aspects depending on the patient classification:

1. Those aged over 60 years showed significantly more elevated levels of cystatin C than the others (*p* = 0.049; Spearman rank correlation coefficients, *r* = 0.260).
2. Those who had advanced disease with extra-nodal involvement also had higher levels of cystatin C compared to those without extra-nodal disease (*r* = 0.573, *p* = 0.005).
3. Moreover, high cystatin C levels were correlated significantly with high serum LDH level, with a rank correlation coefficient *r* = 0.325 (*p* < 0.013).
4. We did not observe any difference in cystatin C levels among patients according to performance status (*r* = 0.056, *p* = 0.763), or disease stage (*r* = 0.107, *p* = 0.491). Lastly, we found that cystatin C levels could not correlate with IPI score (*r* = 0.0874, *p* = 0.604).

#### 3.2.2. Cystatin C, Response to Treatment and Outcome

Two-year overall survival (OS) and disease-free survival (DFS) of all patients was 91.4% and 87.9%, respectively. Considering the results obtained from factors associated with serum cystatin C levels, we evaluated whether serum cystatin C could be a powerful predictor of disease response. Five patients were excluded from the response analysis, since they died early during the course of disease before starting therapy. Using a cut-off value of 4.2877 for cystatin C, determined by ROC curve analysis (Figure 3b), two sub-groups with significant altered response to treatment were detected. Patients whose serum levels of cystatin C were less than or equal to this value were those who achieved response; and those with serum levels higher than the cut-off value showed no response. The probability that the test is significant in detecting treatment outcome of patients with NHL is 34.1% while the specificity is 88.9%.

**Figure 4:**
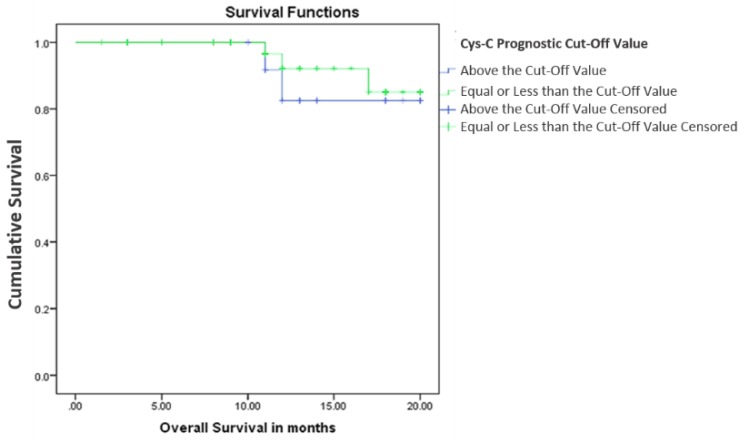
Overall survival according to the serum cystatin C prognostic cut-off value. Cum Survival: cumulative survival.

**Figure 5:**
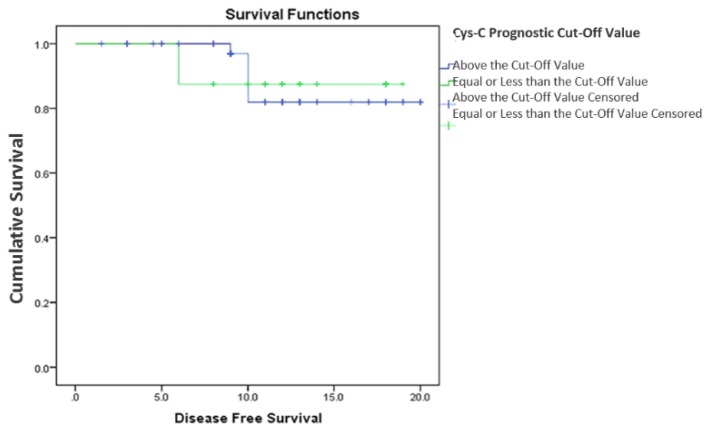
Disease-free survival according to the serum cystatin C prognostic cut-off value. Cum Survival: cumulative survival.

Subsequently, we analyzed the survival according to two sub-groups divided according to the prognostic cut-off value of serum cystatin C levels. Only five patients died during the observation period, therefore OS between the two cystatin C subgroups was not statistically significant (2-year OS: 93.2% vs 85.7%, *p* = 0.612; Figure 4). Furthermore, patients with cystatin C levels equal to or lower than 4.2877 ng/mL at diagnosis showed no significant difference in DFS when compared to those with cystatin C levels higher than the cut-off value (2-year DFS: 88.1 vs 87.5, *p* = 0.843; Figure 5).

**Figure 6:**
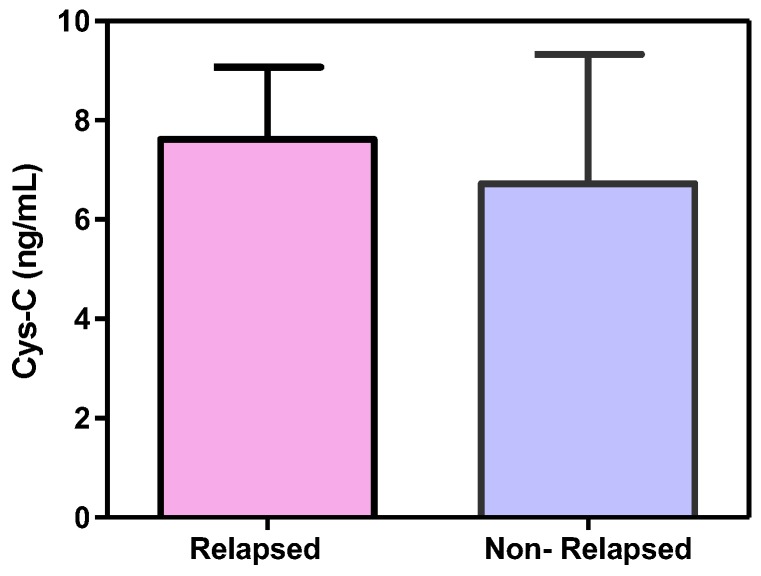
Bar chart shows the difference in serum cystatin C levels among relapsed and non-relapsed diffuse large B-cell lymphoma patients.

Patients were analyzed with respect to relapse of disease in two subsidiary groups where seven patients showed relapse and 37 showed no-relapse; analysis resulted in higher serum cystatin C levels in relapsed patients but of no significant difference versus non-relapsed patients (*p* = 0.167, mean ± SD 7.906 ± 1.460 and 6.738 ± 2.6, respectively; Figure 6).

**Figure 7:**
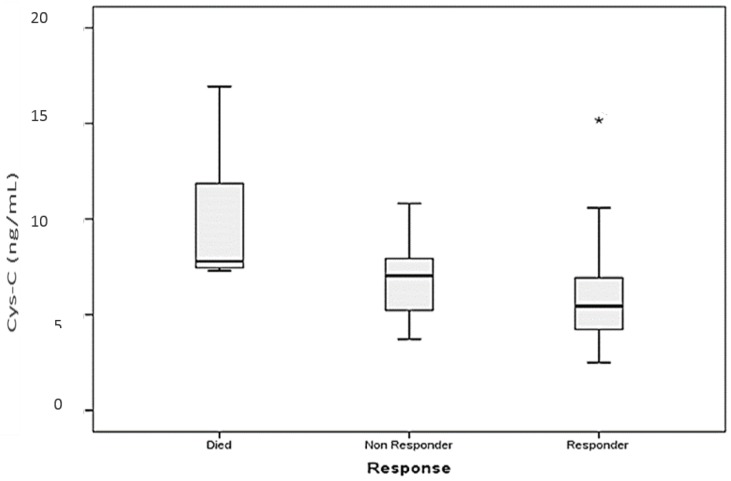
Box plot shows the difference in median and ranges of serum cystatin C (Cys-C) levels among responders, non-responders and patients who died. * Significant different (*p* < 0.05).

Furthermore, patients who died early due to aggressive disease or high tumor burden had a significantly higher median serum cystatin C level than the initial level of cystatin C for patients who achieved response after chemotherapy (7.792, range: 7.307–16.93 versus 5.456, range: 2.49–15.171 ng/mL, *p* = 0.013; Figure 7).

## 4. Discussion

Much attention has been given recently to the identification of prognostic features in NHL. Several scoring systems have been designed and are currently used to define prognosis and, in some cases, to guide therapy. Perhaps one of the most powerful of these systems is the International Prognostic Index [23]. However, as more information becomes available on the pathogenesis of these tumors, the biologic factors that influence prognosis may be better understood. This is the leading study to analyze the influence of serum cystatin C level—showing sensitivity and specificity—on the disease-free survival and overall survival probability of DLBCL patients; DLBCL denotes the most common subtype of NHL, it is accounting for 30%–40% amongst lymphoma cases [1,2].

A previous report suggests the presence of high levels of cystatin C in the sera of patients with B-NHL compared with healthy controls [21]. In agreement with these results, we have confirmed the finding of high serum levels of cystatin C in patients with DLBCL compared to healthy controls. We have further demonstrated that increased levels of cystatin C in pretreated patients were decreased once those patients attained remission after treatment with chemotherapy, proposing that cystatin C levels are allied to tumor burden and could be valued and useful in monitoring the disease response.

In their study on 52 patients with B-cell lymphoma (41 aggressive lymphomas and 11 indolent lymphomas), Softić et al. [25] demonstrated a significant reduction of cystatin C levels with disease remission after the third cycle of chemotherapy, and that aggressive lymphomas almost completely contributed to the statistical significance of the complete sample. Whether it is due to a specific mechanism in aggressive lymphomas, not present in patients with indolent lymphoma, which contributes to decreased levels of cystatin C in disease remission, could not be precisely determined due to the small sample of patients with indolent lymphomas who enter remission after chemotherapy (*N* = 6) [25]. Along this line, a more elevated serum level of cystatin C has been also detected in patients with colorectal cancer and advanced melanoma than in patients with primary melanoma and healthy controls [18,20].

The optimum cut-off level that yields the finest classification of patients according to response in two main groups was noticed at 3.9829 ng/mL. We found that high levels of cystatin C, above the cut-off value, were correlated negatively with response to therapy. These results recommended that it was valuable to examine whether the negative or poor prognostic impression of cystatin C levels on response was due to its relation to the expression of further poor prognostic markers. In this regard, we compared the levels of cystatin C with established biochemical and clinical parameters.

The IPI is the widely accepted prognostic index for patients with aggressive lymphoma [23]. Our results show no correlation of cystatin C with the IPI, although the levels of the inhibitor were associated with two individual factors that are included in the IPI: age and serum LDH activity. In this patient population, serum cystatin C was significantly higher in patients over 60 years of age compared with those under 60 years of age. Elevated levels of cystatin C in patients over 60 years of age may be the result of weakening kidney function due to aging [26]. However, it should be noted that all the patients involved in the study had normal serum creatinine. We assumed that the serum cystatin C level and its correlation with age could not be attributed to a solitary factor such as physiological alteration, but to additional aspects such as effects of disease comorbidity. The cause behind the elevation in cystatin C levels in sera and age needs to be elucidated. Cystatin C levels were also significantly elevated in patients with elevated LDH activity compared to those with normal LDH activity.

Our study also revealed that cystatin C, at the time of diagnosis, correlates with extra-nodal disease. These results suggest the possibility that there is a specific mechanism in extra-nodal infiltration that drives the increased secretion of cystatin C in body fluids. Moreover, cystatin C is produced by all nucleated body cells, so the correlation of the inhibitor level and extra-nodal disease can be the result of the general response to the increased proteolytic activity. In malignancy, a misbalance among cysteine proteases and their inhibitors, accompanied by the metastatic phenotype of the tumor cell, is believed to promote the invasion of the tumor cell and metastasis [27]. Hence, the malignancy spread might be supposed to affect the levels of cystatin C.

In our study, there was no significant difference in the serum levels of cystatin C when comparing advanced to non-advanced disease, which was similar to the results in 39 lung cancer patients reported by Ohara et al. [28]. Different investigators stated that the level of cystatin C is influenced by the extent of the tumor, however, they were not able to find out if the significant correlation between cystatin C levels in sera and tumor burden is reliant on the primary site of the cancer [29]. This discrepancy could be due to the dissimilar biological features between diverse tumor types, and the probability that the role of cystatin C might vary according to type of cancer, cause, and site in addition to stage of cancer.

We have further demonstrated that there is no correlation between pretreatment cystatin C levels and presence of B symptoms. This is consistent with the results from Softić et al. [25], who found no correlation between cystatin C and B symptoms or between the inhibitor level and any of the acute phase reactants such as interleukin-6 or C-reactive protein (CRP), which was expected since the presence of B symptoms is associated with elevated inflammatory proteins including CRP and cytokines.

Mulaomerovi´c et al. [22] revealed more elevated serum cystatin C levels in patients with B-NHL who experienced relapse than those who did not. Hence, they considered cystatin C as a potential marker for relapse in patients with non-Hodgkin B-cell lymphoma. In contrast, we found that serum cystatin C levels have no influence on disease-free survival probability. Comparable trends were found when studying the effect of cystatin C level on overall survival. However, this analysis may be limited by the small number of events and short follow up duration. Only five patients died and seven patients relapsed during the observation period. Analysis of the relapsed group versus the non-relapsed one showed no significant difference; this may be because of the small number of patients who showed relapse. Therefore, we believe that further investigation including a larger number of patients and longer follow up will be required to confirm these findings.

The fact that cystatin C did not correlate with the IPI raises the possibility that it might independently add to the power of the IPI in predicting survival. Although the correlations with survival are compromised by the short duration of the study, and the low number of events, it should be examined whether combining the IPI and cystatin C makes a model that is more predictive than the IPI alone.

In summary, serum cystatin C levels are significantly higher in DLBCL patients than in healthy controls. Moreover, we explored cystatin C levels in DLBCL patients, as a promising biomarker for monitoring response to therapy. On the contrary, it bears no prognostic significance regarding survival.

## Figures and Tables

**Table 1:** Main clinical characteristics of 58 patients with diffuse large B-cell lymphoma (DLBCL).

Variable	No. of Patients (%)
Age (years)	
<60	38(65.5)
≥60	20 (34.5)
Male sex	25 (43.1)
Performance status ^a^	
<2	39 (67.2)
≥2	19 (32.8)
Ann Arbor stage	
I/II	8 (13.8)
III/IV	50 (86.2)
B symptoms	28 (48.3)
Extra-nodal involvement	20 (34.5)
High serum lactate dehydrogenase (>ULN)	48 (82.8)
International prognostic index group	
Low (0–1)	2 (3.4)
Low–intermediate (2)	8 (13.8)
High–intermediate (3)	22 (37.9)
High (4–5)	26 (44.8)

^a^ European Cooperative Oncology Group criteria. ULN, upper limit of normal
